# Combining social and private information: How ants use pheromones and learnt cues to navigate

**DOI:** 10.3758/s13420-025-00697-w

**Published:** 2026-02-25

**Authors:** Cody A. Freas, Cornelia Buehlmann, Marcia L. Spetch

**Affiliations:** 1 Research Center on Animal Cognition (CRCA), Center for Integrative Biology (CBI), CNRS, University Toulouse III-Paul Sabatier, Toulouse, France; 2 School of Life Sciences, University of Sussex, Brighton, BN1 9QG UK; 3 Department of Psychology, University of Alberta, Edmonton, AB Canada; 4 School of Natural Sciences, Macquarie University, Sydney, NSW Australia

**Keywords:** Multimodal interactions, Collective movement, Trap-lining, Hymenoptera, Olfactory, Orientation, Pheromones, Navigation

## Abstract

Ants exhibit remarkable navigational abilities, flexibly integrating private information such as path integration and learnt visual cues with social information in the form of trail pheromones. Far from being simple or rigid directional signals, pheromone trails serve diverse, context-dependent roles in navigation. For instance, they can act as scaffolds during early foraging trips, tethering naïve ants to the nest while they acquire spatial knowledge. Pheromones also function as reassurance signals, confirming that a forager is on the correct path, and as a fail-safe when other cues become unreliable. Additionally, they appear to support multi-vector way-pointing through gating part of the path integrator expression, enabling ants to segment complex routes and to leave and re-enter trails. The regulation of pheromone laying itself is also influenced by private information streams, including internal state, prior foraging success, and navigational memory, highlighting the nuanced interplay between individual experience, environmental cues, and social signals. Together, these findings reveal the trail pheromone is not merely a recruitment or directional signal, but is an integral component of a sophisticated, multimodal navigational system, interwoven with private memories and the individual’s internal state to support flexible navigation.

## Introduction

A tiny ant with a brain 600 million times smaller than that of a human, carries a piece of food while travelling on a busy trail of ants leading back to its colony. Someone watching this behaviour might assume the ant is doing nothing more than following other ants or a chemical trail as it returns home. There is indeed evidence that ants sometimes follow other ants (Franklin & Franks, 2012; Franks & Richardson, 2006) and that chemical trails formed by deposition of pheromones play an important role in guiding ant navigation (Czaczkes et al., 2012, 2013, 2015). Recent experimental work, however, has revealed that the navigational processes associated with the trail pheromone are far more flexible and sophisticated than one might assume from simple observation. Here, flexibility refers to the dynamic nature of navigational behaviour, with ants able to shift the weighting given to private information and social signals, modulating their behaviour in response to changing conditions or conflicting information. Moreover, as we highlight in this review, ant navigation can involve an adaptive interplay between using social information from other ants, and private information based on an individual ant’s perception and experience.

As previously discussed by Dr. Suzanne MacDonald (MacDonald & Ritvo, 2016), to whom this special issue is dedicated, experimental studies of comparative cognition “in the wild” not only expand the range of species that can be studied and questions that can be addressed, but they also allow investigation of cognitive processes in ecologically valid tasks and can further our understanding of how evolution and ecology shape cognitive processes. Although laboratory studies typically offer the most rigorous experimental control, for studies of spatial cognition and navigation, field research offers the advantage of scale and allows the behavioural processes to be studied in the natural environment in which they typically occur. Ant navigation is particularly amenable to experimental field research for several reasons. Firstly, because of their small size and scale, it is often feasible to follow an ant for its full navigational journey, and we can also track individuals throughout their life. Secondly, many of the environmental cues or sensory inputs can be manipulated in the field. Thirdly, findings from controlled laboratory studies can be complemented by studies from the field to more fully understand mechanism and function (e.g., Barrie et al., 2024; Gruter et al., 2011). Fourthly, behavioural knowledge from field and laboratory studies of ant navigation can be elaborated through neuroscience (e.g., Buehlmann, Wozniak, et al., 2020; Collett et al., 2025; Frank & Kronauer, 2024; Kamhi et al., 2020) and modelling (e.g., Baddeley et al., 2012; Kagioulis et al., 2024; Le Moël et al., 2019; Reddy et al., 2022; Wystrach et al., 2020). Finally, because ants are social insects, they provide an excellent model for understanding how navigation is accomplished using both social and private information streams (Barrie et al., 2024; Middleton et al., 2018).

Our review focuses on the interplay between collective information and private information in ant navigation and specifically addresses when, why and how ants use social pheromone information as part of their toolkit for foraging and navigation. It has long been known that ants create chemical trails by depositing pheromones, using some kind of deposited odour to orient, and that this orientation is stopped when the trail is wiped away (Bonnett, 1779). More recently, these observations were experimentally explored, leading to research conducted over several decades showing that pheromone trails affect foraging behaviour (Wilson, 1962). Much of this research has focused on how pheromones recruit other ants to sources of food and the rules that govern when and how much pheromone is deposited or the probability that an ant will be recruited to that source (see review by Czaczkes et al., 2015). Early work identified a few simple rules: pheromone is deposited by fed ants; amount deposited is based on the quality of the resource; probability of leaving the nest and following a trail is based on the amount of pheromone. The interaction of these simple rules (i.e., trail reinforcement via pheromone concentration and an individual’s response to this trail strength) has been proposed to generate the complex organisation of ant-foraging networks (e.g., Beckers et al., 1993), though this fails to fully capture the range of behavioural complexities observed on ant trails. Experimental work has identified additional complexities showing not only that pheromones serve multiple roles in recruitment, but that they can also modulate trail-following intensity, encode information about food quality or reliability, and be context dependent. Furthermore, ants often integrate trail pheromone information with private information (i.e., motivational state, previous foraging success, and spatial memory), leading to more flexible and adaptive navigational behaviours (Hölldobler, 1971). Recent studies suggest that integrating social and individual information streams allows ants to flexibly respond to dynamic environments and is vital for recruitment to food sources, scaffolding of learning, providing a backup mechanism during uncertainty (e.g., low light), reassurance of cues along the route, or way-pointing for route segmentation. The combined use of social and individual information can result in a highly sophisticated system that is adaptive, efficient and flexible.

## Concurrent use of private information and social signals

Recent research on wood ants (*Formica rufa*) provides an excellent illustration of concurrent use of both private cues and the pheromone signal as well as how laboratory studies and field research complement each other. Wood ants forage in densely cluttered woodlands where they travel along shared odour trails to trees that are up to 100 m away from their nest to get honey dew from aphids (Domisch et al., 2016; Rosengren et al., 1979; Rosengren & Fortelius, 1987). We know from research performed in the laboratory that *F. rufa* ants use a range of sensory cues for navigation including visual (e.g., Buehlmann et al., 2016; Graham et al., 2003; Harris et al., 2005; Lent et al., 2010) and non-pheromone olfactory cues (Buehlmann, Aussel, et al., 2020). Hence, these ants are experts in using individually acquired spatial knowledge in laboratory scenarios when there is no social odour trail available. Our understanding of the natural navigation in these ants, however, remains sparse. Recent behavioural experiments in the ants’ natural habitat have investigated if these ants also acquire individual memories for habitual routes when they are travelling back and forth along odour trails in visually complex woodland habitats. Indeed, tracking entire natural homing paths of individual wood ant foragers revealed that these ants follow idiosyncratic routes, requiring the learning of environmental features while travelling back and forth along collective odour trails (Barrie et al., 2024). Furthermore, some learnt route information is quite stable over time, likely allowing foragers to return to previously successful sites. Site memories have been shown to persist through months of winter hibernation, possibly granting colonies quick access to known resource patches in the spring (demonstrated in *Formica uralensis*; Salo & Rosengren, 2001). Using visual and olfactory information simultaneously (Barrie et al., 2024; Buehlmann, Aussel, et al., 2020; Rosengren, 1971) likely requires movement patterns that facilitate the sampling of multiple sensory modalities. For example, oscillatory (zigzag) paths allow ants to alternately sample visual scenes and olfactory gradients (Hangartner 1967). Lateral oscillations or zigzag paths can amplify left–right olfactory contrasts by changing antennal placement, enabling steering toward the stronger pheromone signal. These oscillations are also highly important for visual sampling in ants (Clément et al., 2023), suggesting oscillations may underlie both visual and pheromone information sampling simultaneously. Wood ants, like many ant species, exhibit such oscillatory movement when navigating using visual cues (e.g., Graham & Collett, 2002; Lent et al., 2010, 2013). Although these studies were not conducted on pheromone trails, Lent et al. (2013) proposed that the phase-dependent control of these zigzag paths could also support odour trail following.

This raises the possibility that the structure of the walking path itself may facilitate the integration of visual and olfactory information. However, direct experimental evidence linking path structure to enhanced multimodal integration remains limited and warrants further investigation.

## Pheromone as a scaffold for learning

If resources around an ant nest are scattered and unpredictable (e.g., dead insects as food), the benefits of recruiting others along pheromone trails may be limited for small, dispersed items, and solitary foraging may often suffice, particularly in desert environments, where pheromones can quickly disperse, as observed in thermophilic desert ant species (Freas & Cheng, 2022; Muser et al., 2005; Wehner, 2003, 2020). Nevertheless, recruitment can still play an important role for larger or clumped food items. Solitary foragers rely primarily on private information to navigate, though social information (e.g., colony odors and the nest’s CO_2_ plume) especially around the nest can aid in pinpointing the nest (Buehlmann et al., 2012; Freire et al., 2023).

In other ants, such as the red harvester ant *Pogonomyrmex barbatus*, group foraging is regulated through social feedback (i.e., returning scouts), indicating suitable foraging conditions for mass recruitment (Greene & Gordon, 2003). If resources are clumped, reasonably stable, or too large or plentiful for an individual to collect, recruitment to the resource substantially enhances the efficiency of foraging. Many species of ants use pheromones for recruitment to such food sites, and this has been extensively studied. Czaczkes et al. (2015) provide an excellent review of much of this literature. In the current review, we highlight studies that illustrate the sophistication and flexibility of pheromone use and how these social information signals act together with privately learned cues.

### Effects of personal experience on the use of pheromone trails

Pheromone trails and private information can be used synergistically during route formation (Evison et al., 2008); however, the weighing of these information streams has been shown to depend on the ants’ foraging experience. Chemical trails allow ants that are inexperienced or unfamiliar with an environment to successfully reach a feeding site or the nest. These trails, similar to path integration (a process in which insects continuously update their position relative to the nest by integrating distances and directions traveled using an odometer and celestial compass), provide a scaffold allowing ant foragers to learn individual routes while being safely connected to the nest (Collett, et al., 2003; Hangartner, 1969; Harrison et al., 1989). Trail-following ants are thus strongly guided by pheromones when they are naïve and they need to acquire individual route memories; however, various experiments have shown that experienced ants often prioritise individually learnt cues when these are experimentally set in conflict (e.g., Grüter et al., 2011; Hangartner, 1969; Harrison et al., 1989). Yet, weighting between the pheromone signals and memory-based information depends on both the species and the relative strength or reliability of the information streams (von Thienen et al., 2016).

In experimental field studies on the giant tropical ant *Paraponera clavata*, researchers placed a Y-maze along a trunk trail, offering sucrose at the end of one branch and water at the end of the other (Harrison et al., 1989). Paper was laid on the floor of the maze and ants deposited pheromone when returning from the sucrose branch. On tests, the paper was switched so that the side containing the pheromone no longer pointed in the correct direction according to visual cues (i.e., landmarks). Most inexperienced ants travelled along the pheromone-paper arm, suggesting the pheromone was effective in recruiting naïve ants. However, most experienced ants travelled in the direction they had previously found food, even though it had no pheromone. Thus, the pheromone signal was used until the visual cues were learned and then those visual cues dominated when experimentally put in conflict. A subsequent set of tests in which each information type was blocked provided further evidence that experienced ants can use either, but prioritise visual cues.

In a similar study on the Australian trunk-trail-forming ant *Iridomyrmex purpureu*s, it was shown that ants switched from following pheromones to prioritising learned information as they gained experience (Card et al., 2016). These ants form trunk trails to regularly visit long-lasting food sources (Greaves & Hughes, 1974; Nel, 1965). Card et al. placed a paper-lined Y-maze partway along a trunk trail with one branch leading to food. Conflict tests, similar to those of Harrison et al. (1989), showed that pheromone trails facilitated initial recruitment, whereas visual cues dominated once the route was familiar. In another set of tests, ants allowed to collect food were displaced to an unfamiliar location lacking both pheromone trails and familiar visual cues. Their initial homeward orientation indicated the use of path integration. The ants did not travel the full distance home, and the distance covered decreased with longer distances from the nest. The researchers did not test how pheromone cues might interact with path integration. However, other studies show that ants do not fully walk back their path integration vector when visual surroundings are unfamiliar (Buehlmann et al., 2011; Freas et al., 2021; Narendra, 2007; Wehner & Srinivasan, 1981); therefore, the lack of familiar visual cues may have influenced the behaviour of *I. purpureus* in these tests.

## Informational content controls the use of private cues and social signals

One reason for prioritising memories over the pheromone is that the private memories may provide greater informational content (Czaczkes et al., 2019). Specifically, individual memories formed during foraging may encompass both spatial information required for successful navigation and information about the quality of the food, whereas pheromone trails may convey less detailed information about food quality. Although the quantity of pheromone deposited varies with food quality, there is considerable variability across individuals in the amount of pheromone deposited for a specific quality of food, making this a noisy signal for communicating differences in food quality. Czaczkes et al. (2019) investigated how the richness of social information influences ants’ use of private versus social information by making the social information carry additional cues about food quality. They created a conflict in a Y-maze between individually acquired route information (private experience) and the pheromone (social information). More ants followed the pheromone trail when the researchers added a small droplet of sucrose to the trail, thereby providing extra information that indicated a higher-quality food. In a second experiment, information about food quality was given via nest mates that had been fed either a weak concentration of sucrose (matching the test ant’s prior experiences), or a much stronger concentration of sucrose. More test ants that had contact with nestmates fed the strong sucrose followed the pheromone trail on the Y-maze. These results suggest that the directional information provided by a pheromone trail is more likely to be followed when those cues provide additional, reliable information about food quality that surpasses their own previous experience.

A complementary finding is that following a pheromone trail increases when visual memories offer less certainty. Jones et al. (2019) varied light level as ants were learning to find a food source in a T-maze. When the light level was reduced, ants deposited more pheromone. Moreover, on conflict tests when the pheromone conflicted with route memory, the number of ants choosing the pheromone side increased if they were tested in the lower light condition. Hence, these ants relied more strongly on pheromones when visual cues were less reliable due to a lack of light.

One interesting question regards the richness of the informational context of the pheromone signal itself. There is evidence that Pharaoh’s ants (*Monomorium pharaonis*) may deposit a negative pheromone on return from non-rewarded locations, as a “no-entry” signal (Robinson et al., 2005). Using a Y-maze, researchers placed paper from a branch that had led to non-reward against neutral paper on a maze that previously had both branches rewarded. The ants were significantly more likely to choose the branch with the neutral paper, and significantly more likely to turn back to the nest when in contact with the paper from a previously unrewarded branch. These results suggest that pheromones can not only be used to recruit ants to profitable locations but also to deter ants from travelling to unprofitable locations. However, subsequent attempts to replicate the presence of the no-entry signal at the trail bifurcation have been unsuccessful (Troitino 2017; Zanola et al., 2025), and suggest that private aversive memory formation may instead play a primary role in the abandonment of punished trails (Zanola et al., 2025).

Through behavioural experimental research and mathematical modelling, Dussutour et al. (2009) showed how the flexible use of two different pheromones may enhance the foraging success of an invasive ant species, the big-headed ant *P. megacephala*. They demonstrated that these ants deposit a long-lasting pheromone that recruits exploration as well as a shorter-acting pheromone that recruits exploitation when a good food source is found. The exploration pheromone enhances exploitation whereas the exploitation pheromone suppresses exploration. The researchers’ mathematical modelling suggests that the combination of these two functionally different pheromones allows the ants to quickly mobilise when food is found as well as to adapt quickly to dynamically changing environments. They speculate that this provides a powerful foraging strategy that may contribute to this species’ success in dominating native species when introduced into new areas. Although this example does not involve private information, it does illustrate how nuanced pheromone signalling alone can produce adaptive collective behaviours.

On the other hand, it was reported that although garden ants (*Lasius niger*) can learn to change their behaviour toward pheromone trails, there are limits to their flexibility (Wenig et al., 2021). The researchers trained ants on a Y-maze where one arm led to punishment (i.e., quinine solution and/or shock) and the other arm led to sucrose. The punished side randomly varied across trials so that location memories could not be used reliably. When the punished arm contained a pheromone trail, choice of the pheromone arm decreased to chance level, but did not go below chance. This suggested that the ants learned to stop following the pheromone trail (i.e., learned to ignore it), but they did not learn to actively avoid it, which would require reversing its meaning and treating it as aversive (i.e., punished choice falling significantly below chance level). In a control experiment, they showed that the ants could learn to actively avoid a non-pheromone odour placed on an arm that led to punishment.

This asymmetry may reflect a fundamental constraint in how pheromone cues are cognitively processed. Pheromones are evolutionarily stable signals that typically indicate beneficial paths, and may be interpreted as inherently neutral or positive. As such, ants may lack the ability to reclassify them as aversive, even when paired with punishment. This limitation is particularly evident in socially navigating ants, where trail fidelity supports collective foraging efficiency. In contrast, solitarily navigating ants rely more heavily on private information and exhibit greater flexibility in abandoning unreliable or punishment associated cues. The inability to avoid pheromone trails may therefore reflect a trade-off between the flexibility of private information use and the consistency of social signals.

Taken together, these results suggest that the informational content of social and private cues can be an important determinant of which cue ants prioritise, as well as when and to what extent they rely on these cues (Robinson et al., 2005; Troitino, 2017; Zanola et al., 2025). At the same time, the apparent inability of some species to actively avoid locations marked by pheromones, even when signals are outdated or punished, suggests a limit to their flexibility once a pheromone is laid (Czasckes et al., 2024).

## Pheromones as reassurance signals and fail-safes

### Private information resolves pheromone trails’ lack of polarity

Although the pheromone is often used to navigate between the nest and food sources in trail-following ants, there are limitations on the pheromone signal, and research has shown how private information can be used together with pheromones to adapt to new situations, and to provide navigational behaviour that is more efficient and fail-safe. One such limitation is that pheromone trails lack inherent polarity, meaning that in isolation, the pheromone trail does not signal whether a forager is travelling nestward or foodward. Thus, ants must rely on private cues to resolve this ambiguity.

**Fig. 1:**
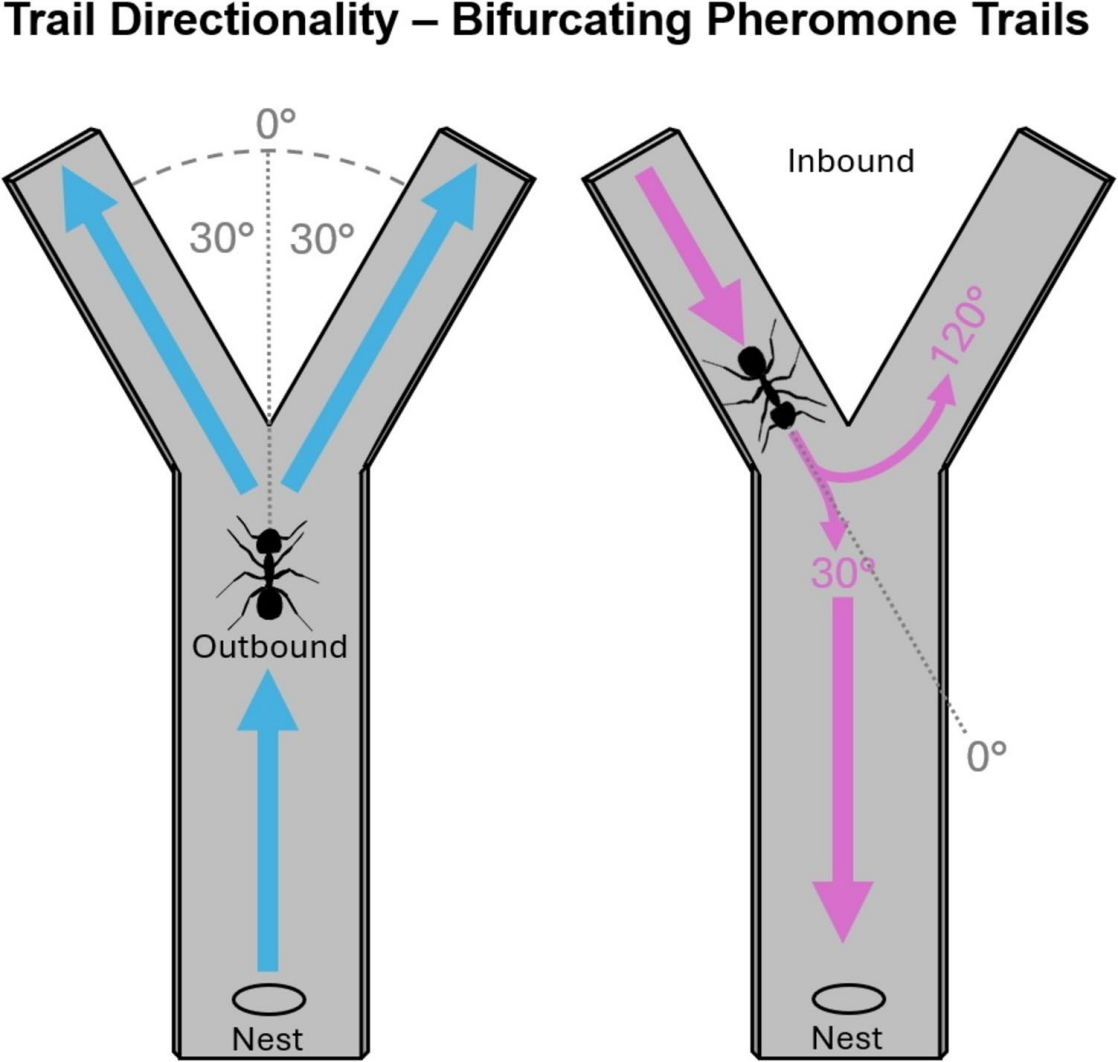
Geometry of bifurcating pheromone trails provides polarity information to both outbound and inbound ants. Bifurcating trails will often branch in the outbound direction in a ~60° angle (blue arrows), resulting in a ~30° turn. Inbound ants (pink arrows) approaching the same junction face two options: a 30° turn (leading correctly back to the nest) or a 120° turn (leading away from the nest). By consistently selecting the smaller turn (30°), ants maintain correct orientation within bifurcating trail networks

Attempts to discover an inherent trail polarity have focused on concentration gradients which could, in theory, provide an orientation signal. However, behavioural evidence for inherent polarity of pheromone trails is lacking in ants (Carthy 1951; Freas & Spetch 2021, 2023b; Wetterer et al., 1992), at least in non-bifurcating trails (Jackson et al., 2004). In branching trails, arrangement of the branching network itself can encode such information via the branch angle, which is commonly ~60° across many species (Acosta et al., 1993; Fig. 1). One such species, Pharaoh’s ants (*Monomorium pharaonis*), can use the asymmetry of this trail geometry to orient correctly when reaching branch connections, turning towards the trail representing the least angular distance from their current heading, which corresponds to the nest direction for inbound ants (Jackson et al., 2004). Importantly this turning dynamic relies on private information, with both the ant’s internal motivational state (e.g., inbound vs. outbound) and private memories (e.g., path integration and/or the visual landmarks) dictating turning behaviour in response to the geometric characteristics of the trail network (Fig. 1).

For ants moving along non-branching trails, private directional cues resolve the lack of directional polarity on the pheromone trail. Inbound Leaf-cutting ants (*Atta cephalotes*) will move along a trail marked with pheromone in line with the surrounding visual cues, even when the pheromone is rotated 180°, causing foragers to travel ‘outbound’ according to the pheromone trail (Wetterer et al., 1992). It was not specified, however, whether these visual cues were terrestrial landmarks, path integration cues, or both.

Desert harvester ants (*Veromessor pergandei*) do not attend to terrestrial views (landmarks), relying solely on interactions between the pheromone and their path integrator to make navigational decisions (Freas, Congdon et al., 2019a, 2019b; Freas, Plowes et al., 2019a, 2019b; Freas & Spetch, 2021). When the pheromone trail is rotated 180° beneath foragers travelling to the nest, these individuals continue to follow their path integrator, despite now travelling outbound according to the pheromone. These streams work together, with the presence of the pheromone promoting vector-based orientation, even when a forager had recently run off their vector. When inbound ants were collected at the nest (no remaining vector) and placed back on the pheromone trail, they still oriented in their recent vector direction, likely reloading a vector memory of the recent trip (Collett & Collett 2000; Freas & Spetch, 2021). Together these behaviours make ecological sense, as foragers that have little to no remaining vector, yet are still on the pheromone trail, likely have not yet reached the nest.

In garden ants (*Lasius niger*), learning the surrounding terrestrial scene can provide individuals with directional information on the trail. Minoura et al. (2016) rotated the visual scene around ants as they navigated to the nest when on the pheromone trail. Homing ants responded by turning on the trail, travelling in the outbound trail direction when it aligned with the inbound visual scene. Similar results have been shown in Western thatching ants (*Formica obscuripes*), which rely on both the learned terrestrial scene and their path integration system to orient correctly on the pheromone trail (Freas & Spetch, 2023a, 2023b). When the pheromone trail was rotated 180°, placing the trail in conflict with either the path integrator or the inbound terrestrial scene, *F. obscuripes* foragers continued to travel according to the visual cues rather than following the rotated pheromone trail.

Private cue use has also been demonstrated in *Pheidole oxyops*, where short-lived pheromone trails act as a passive net, recruiting both newly emerging foragers from the nest and intercepting foragers already active outside (Czaczkes & Ratnieks, 2012). These trails did not need to extend all the way to the goal location; instead, foragers encountering them were able to reorient correctly, suggesting they combined the pheromone trail with private information (path integration or visual cues) to determine the appropriate direction.

Taken together, these findings demonstrate that ants can determine travel direction along a pheromone trail using learnt environmental cues. Moreover, they are capable of simultaneously integrating three distinct information sources, the pheromone trail as a directional axis, visual cues to establish polarity, and their internal state, to make navigational decisions when encountering a trail.

### Memory-guided orientation at the trail edge

Although the structure of the pheromone trail lacks inherent polarity, ants use an interplay of olfactory sampling and their private memories to maintain orientation as well as avoid leaving the trail. Ants use their two antennae, conducting bilateral comparisons to conduct tropotaxis (comparisons of pheromone strength across the two antennae, orienting towards the higher concentration), assessing the pheromone’s strength on each side of its path to inform where to orient (Hangartner, 1967; Schone, 1984; Draft et al., 2018). Carpenter ants have been shown to conduct dynamic antennal movements in line with their current orientation behaviour indicating specialised olfactory sampling and tropotaxis (Draft et al., 2018). Similar bilateral comparisons occur in bifurcating trails and can direct ant navigators (Perna et al., 2012). Here, Argentine ants were shown to quantify the local differences between pheromone concentrations on the two sides of their body to determine the magnitude of the turning angle, illustrating that bilateral discrimination of local pheromone concentrations via antennae produces differences in turning.

This olfactory information sampling likely works in tandem with private memories to aid ants travelling on pheromone trails to maintain the correct orientation. Specifically, memory provides the general direction to the goal (subject to noise), whereas behavioural information sampling of the pheromone aids in correct orientation and confirms the ant is still on the trail (Czaczkes et al., 2011; Freas et al., 2021; Wetterer et al., 1992). These cross-modal interactions between information streams and navigational systems are a critical part of how ants and other insects navigate (Barrie et al., 2024; Buehlmann, Aussel, et al., 2020; Buehlmann, Mangan, et al., 2020b, 2020c, 2020a).

Both the terrestrial scene and the path integrator influence how foragers respond when they encounter the trail’s edge. Garden ants will abandon the trail to follow familiar terrestrial scenes when they conflict with trail directions (Minoura et al., 2016). Memories of terrestrial scenes often outweigh other information (e.g., path integrator and the trail) during navigational decisions. A more complex interaction occurs when the trail and celestial compass directionally conflict. The path integrator is known to increase in salience strength along with its distance component when in conflict with other information streams (Buehlmann, Mangan, et al., 2020b, 2020c, 2020a; Heinze et al., 2018; Wystrach et al., 2015). This increased path integrator signal strength means ants will weigh this cue heavily versus the social signals of the pheromone.

In desert harvester ants (*Veromessor pergandei*) the path integrator will dominate orientation decisions, even when in conflict with the pheromone trail. If their path integrator is in 90° conflict with the pheromone trail, the length (and its corresponding salience) of the vector determines whether the ants pass the trail’s edge or not (Freas & Spetch, 2021). When the path integrator is long (i.e., indicating a large amount of the trail lies ahead), ants will readily leave the pheromone whereas ants with short path integrators will hesitate and retreat back into the trail, possibly to avoid overshooting the nest (Freas & Spetch, 2021). Hence, the pheromone acts as a contextual signal to modulate private navigational system use.

#### Backtracking on and off the trail

Backtracking is a navigational backup mechanism, which is widely observed across ants when homing to the nest, yet the exact underlying mechanisms are diverse and linked to foraging ecology. Inbound foragers, having almost reached their nest and displaced to unfamiliar areas, do not engage in systematic search for the nest entrance. Instead of searching, these individuals orient backwards in the compass direction opposite to the path they have just travelled, a behaviour thought to act as a safeguard to prevent foragers from overshooting the nest (Wystrach et al., 2013). In solitary foraging species, backtracking typically requires that foragers are presented with an unfamiliar visual scene, have recently observed the visual scene around the nest, and have a near-zero path integrator state (Wystrach et al., 2013).

Socially navigating ants also backtrack, but the criteria are altered, with the pheromone’s presence playing a critical role in the expression of backtracking. In desert harvester ants (*V. pergandei*), which do not rely on landmarks, backtracking behaviour is dependent on an interaction of the path integrator state being near zero and the ant no longer being in contact with the pheromone trail (Freas et al., 2019a). In trail-following species (*Formica obscuripes*) where landmark-based memories are utilised, both an unfamiliar visual scene and the absence of the pheromone criteria must be present for backtracking to express (Freas & Spetch, 2023a). Thus, the pheromone’s presence acts to reassure foragers that they have not overshot the nest, even when the visual scene becomes unfamiliar and the path integrator is near zero.

## Pheromones as a contextual signal for way-pointing

Beyond providing reassurance in uncertainty, pheromones also serve as contextual signals, supporting way-pointing and route retracing by triggering transitions between distinct vector memories and guiding ants through segmented, non-linear paths (Freas et al., 2020; Freas & Spetch, 2024). In these cases, the pheromone appears to gate the expression of specific vector memories at key points along a route, enabling ants to return home via structured, multi-stage navigation. Thus, the pheromone may be a key part of how these ants form their spatial representation system.

Untangling this phenomenon requires quickly describing the hybrid pheromone foraging structures of some groups of foraging ants. *V. pergandei* are seed collectors that navigate along a shared pheromone trail column. Near the food source, the pheromone column ends and foragers spread out in a fan, searching for seeds solitarily, relying on their path integration system (Plowes et al., 2013, 2014). Once a seed is collected, foragers will navigate back to the pheromone column, a critical non-nest way-point along the inbound route, before heading back along the pheromone trail to return to the nest (Plowes et al., 2019). Similar way-pointing, i.e., homing foragers that return to the column before following the column back to the nest, is observed in other trail-following ants as well (see Flanagan et al., 2013).

**Fig. 2:**
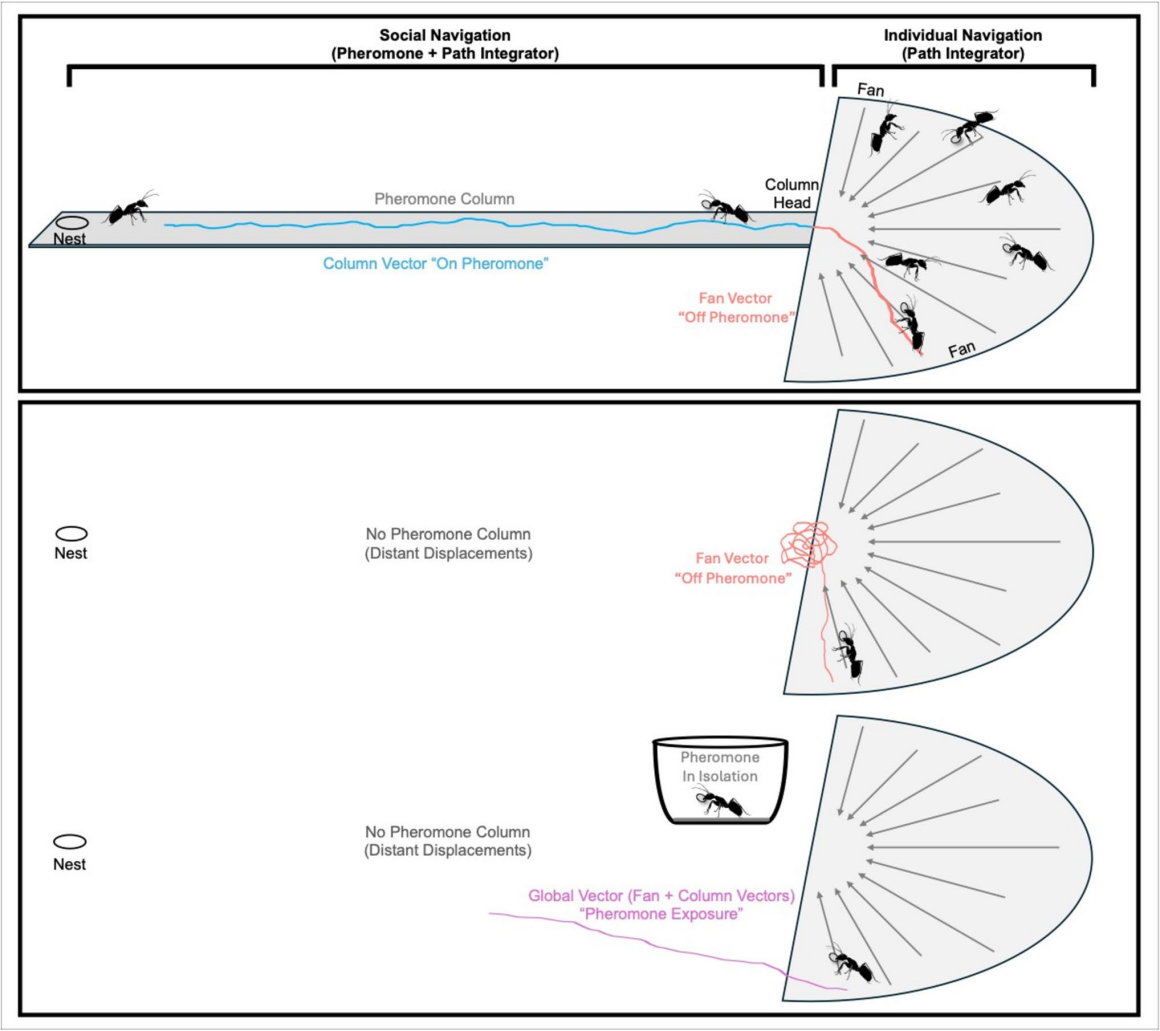
Pheromone functions as a contextual cue supporting way-pointing via transitions between distinct vector memories. **Top row:** In a column-and-fan foraging structure, ants first socially navigate along a pheromone column, then leave the pheromone at the column's head to fan out and collect food. On return, ants follow only a portion of their path integrator, a fan vector (red) back to the column head, then employ a column vector (blue) to reach the nest. These vectors trade off under natural settings, as the fan vector runs off and becomes uninformative at the column head, where the column vector activates and guides the ant home. **Middle row:** If inbound ants in the fan are displaced and fail to reach the pheromone at the end of their fan vector, they search for the column head (red), as the column vector only activates upon pheromone detection. **Bottom row:** Artificial exposure to pheromone while in the fan reactivates the column vector. Importantly this is not a switch where the fan vector is suppressed. Instead, foragers orient using a combination of both vectors or their ‘global vector’ to the nest (pink), suggesting integration of the two vector memories upon pheromone exposure

This multi-stage navigation is managed by interactions between multiple path integration-derived vectors and the pheromone’s presence (Freas et al., 2020), with foragers possessing distinct vector memories, which express separately in light of the pheromone context to enable way-pointing (Fig. 2). A fan vector expresses when ants navigate individually in the absence of the pheromone. This vector memory returns the navigator to the column where ants will begin to search until pheromone contact is restored. At this point, a column vector, which is suppressed until pheromone contact, is expressed, guiding foragers along the trail towards the nest. The column pheromone then acts as a contextual gating cue, defining a non-nest node that triggers expression of the column-origin vector while the ongoing fan vector remains active. The resulting composite, or global vector (fan + column), cannot be elicited by path integration alone; it requires an external categorical signal to gate the coexistence of multiple stored vectors.

Under natural settings, these two vectors would trade off, with the fan vector reaching near zero (thus becoming directionally uninformative) at or near the pheromone trail, where the column vector activates and directs the ant home. Importantly, pheromone contact does not suppress the fan vector, but merely ends the suppression of the column vector. This means that if foragers encounter the pheromone while still possessing a remaining fan vector, they will orient via a combination of the two, suggesting the two vector memories are integrated upon pheromone exposure (Freas et al., 2020; Fig. 2), making this a hallmark of complex and flexible multi-vector navigation.

**Fig. 3:**
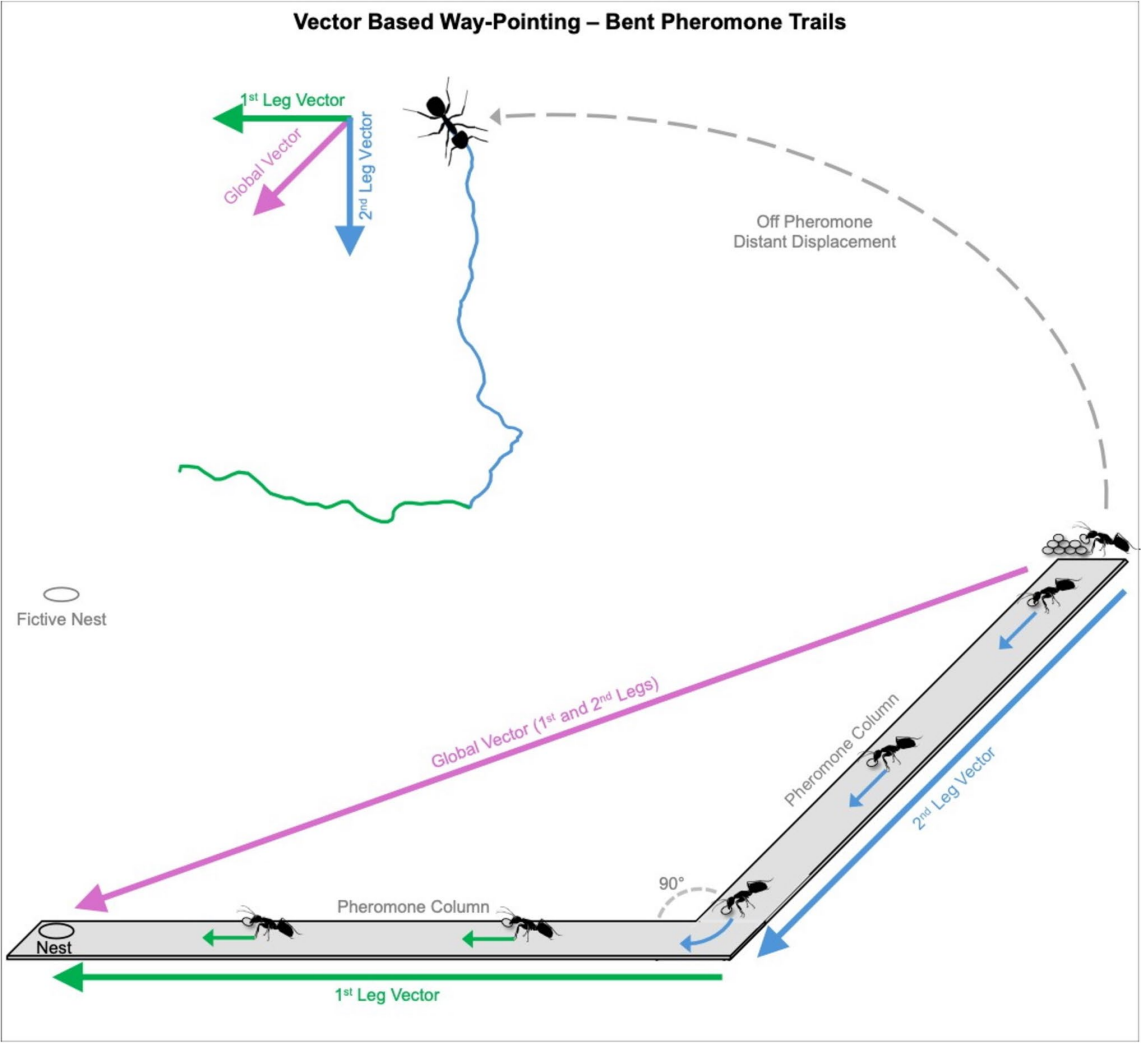
When pheromone trails are bent rather than straight, trail-following ants tend to retrace their outbound path by staying in contact with the pheromone, rather than shortcutting directly (pink) to the nest using their global vector. Foragers achieve this retracing through path integration, attending to each leg segment in sequence, with turns guided by their internal path integrator state. Importantly, when these ants attempt to retrace their route when there is no pheromone present, they seem to switch to the next segment (Leg 1 – blue) to the next (Leg 2 – green) much earlier, distance-wise, than their vector’s true length. The absence of the pheromone promotes early expression of the next vector segment, possibly as a contextual fail-safe signal to prevent foragers from overshooting the turn

Whereas fan and column way-pointing relies on the conditional expression of distinct vector memories with the pheromone as a contextual trigger, some ants have also shown the ability to way-point within the pheromone trail. Here, the pheromone is continuously present, but foragers must follow non-linear, multi-legged paths back to the nest, weaving around bushes, rocks and other clutter (Fig. 3). This would create a problem for a single path integration-based vector representing the full pheromone column, which would direct inbound ants along the straight-line route home rather than retracing the outbound route. Such a strategy would push inbound foragers off the pheromone trail, which is not observed in inbound ants, which instead retrace these winding routes, likely to remain on the trail (Freas et al., 2020; Freas & Spetch, 2024).

When ants’ vector orientation on non-straight trails is tested, via collecting individuals from distinct sites on the trail where the next leg and the nest directionally diverge, ants orient as if they are following only part of their column vector in the direction of the upcoming leg direction rather than the nest (Freas et al., 2019a; Freas & Spetch, 2023b, 2024; Fig. 3). In *V. pergandei*, when non-straight trails with multiple directionally distinct legs are created, foragers attend to each segment in sequence and reorientations are linked to the internal path integrator state. As these retracing behaviours occur in the absence of any familiar cues (i.e., at distant locations), these foragers can switch between multiple vector memories without the need for view following or pheromone-based triggers (Fig. 3). Interestingly, when these ants attempt to retrace their route when there is no pheromone present, they seem to switch to the next segment much earlier, distance-wise, than their vector state would dictate (Freas & Spetch, 2024). This suggests that under these non-staightline routes, the absence of the pheromone promotes early expression of the next vector segment, possibly as a contextual fail-safe signal to prevent foragers from overshooting the turn. This is somewhat like acting as a reassurance signal discussed above, but here it triggers the activation of distinct vector memories rather than a switch from vector orientation to searching.

Waypointing in trail-following ants, both when retracing multi-legged pheromone trails and during multi stage on/off pheromone routes, illustrates a complex link between vector memories representing distinct segments of the route and the pheromone. Here, the pheromone can act as both a contextual trigger for the expression of subsequent trail sections and also likely acts as a contextual scaffold that prevent premature reorientation on non-straight-line trails. This mechanism mirrors trapline foraging in bees, where individuals visit multiple sites in a fixed order using stored vector memories (Buatois & Lihoreau, 2016; Lihoreau et al., 2012; Ohashi et al., 2006). In trail-following ants the pheromone appears to be deeply ingrained into the expression and sequencing of multiple vector memories, potentially underpinning a division of space to support complex navigation.

## How private information shapes signal provision

Up to this point, this review has focused exclusively on how the interplay of private spatial information and the pheromone trail guides navigation in foragers, yet the pheromone is a signal provided by nestmates and, thus, the regulation of its laying can also be shaped by interactions with the layer’s private information. Ants are known to modulate their pheromone laying after navigational errors (Czaczkes et al., 2013) or when environmental conditions change (Czaczkes & Heinze, 2015). Notably, individuals that made an error, then successfully reached food, deposited more pheromone, while outbound ants on their way towards the food source deposited less if they were going to make an error. This pattern is reminiscent of findings in bees (Perry & Barron, 2013), where deposition appears sensitive to confidence in spatial memory.

Spatial memory may also influence where pheromone is deposited. Ants lay more pheromone closer to food sources and further away from the nest (Czaczkes et al., 2024; Devigne & Detrain, 2006), potentially reflecting that odometric or visual memory-based distance estimations are involved. However, ants appear limited in their ability to revise or suppress erroneous trails. For instance, they do not increase pheromone deposition on alternative branches when a marked trail leads to no reward (Czaczkes et al., 2024), and evidence for inhibitory signals like stop pheromones remains inconclusive.

Additionally, ants adjust pheromone deposition based on the discrepancy between expected and actual food quality, increasing or decreasing trail strength accordingly (Wendt et al., 2019). These findings suggest that private information not only guides navigation but also plays a strategic role in shaping the social information landscape for nestmates. Ants appear strategic in how they provision these social signals via the pheromone, with internal state, context and the resource distance all taken into account. Thus, ants appear to have some internal metric of uncertainty for the information they are sharing and adjust signalling accordingly.

Pheromone provision is not limited to an individual forager. During cooperative transport, where individuals must recruit and coordinate nestmates to move oversized food items, ants also flexibly modulate pheromone laying in ways shaped by context and private information. When a longhorn crazy ant (*P. longicornis*) encounters a food item too large to carry alone, it returns to the nest to recruit help, laying a short-lasting pheromone along the way (Fonio et al., 2016). Cooperative transport of a large item poses some interesting challenges. Firstly, the route taken by an individual ant may not be feasible for ants carrying a large load. Secondly, each individual ant carrying the load may have only partial sensory information. Interestingly, many of the recruited ants do not participate in carrying the load but instead help to “direct the way” by laying short bouts of pheromone trails in the direction of the nest. Experimental tests that placed an obstacle consisting of a barrier with a slit large enough for an individual but too small for the load, showed that the carrying ants abandoned the trail and turned and moved perpendicular to the barrier and eventually around it (Fonio et al., 2016). Thus, the carrying ants initially used the pheromone trail but abandoned it when it meant becoming stuck, collectively finding a new path around the obstacle. This behaviour suggests that, in addition to following social signals, these ants must rely on private information, such as proprioceptive feedback during carrying as well as memory of recent movements, when deciding to override the social cue of the trail. Thus, both the ants providing pheromone and those engaged in cooperative transport rely on a dynamic interplay between private information and social signals, enabling flexible and adaptive collective navigation.

## Conclusion

Research stretching back over a century has highlighted the sophisticated navigational toolbox of ants. This ever-expanding body of work paints the portrait of highly complex and adaptive navigational systems that are fine-tuned to each species’ foraging ecology and produce adaptive navigational behaviours. Such behaviours are even more impressive given the small size and limited neural processing power these animals possess and challenging the notion that they simply follow nestmates or chemical trails. This review synthesises growing evidence that trail pheromones are not merely recruitment or directional signals, but integral components of a multimodal navigational system. Pheromones serve as scaffolds for learning, reassurance signals, way-points for vector transitions, and modulators of trail laying ‒ each role dynamically interwoven with private memories, internal states, and environmental context. These behavioural discoveries have advanced our understanding of how small-brained animals solve spatial problems through flexible cue integration, with implications that extend beyond comparative cognition. Beyond biological systems, these efficient solutions to spatial problems have applications in designing autonomous agents and collective robotics, especially for decentralised navigation and decision-making tasks (Mangan et al., 2023; Payton et al., 2001; Webb, 2002).

## Data Availability

Not applicable.
